# Use of microRNA‐encoded peptides to improve agronomic traits

**DOI:** 10.1111/pbi.13654

**Published:** 2021-07-17

**Authors:** Mélanie Ormancey, Bruno Guillotin, Hélène San Clemente, Patrice Thuleau, Serge Plaza, Jean‐Philippe Combier

**Affiliations:** ^1^ Laboratoire de Recherche en Sciences Végétales CNRS/UPS Auzeville‐Tolosane France; ^2^ Micropep Technologies Auzeville‐Tolosane France

**Keywords:** microRNA, peptide, miPEPs

MicroRNAs (miRNAs) are small regulatory RNA molecules (21–24 nt) regulating the expression of target genes at the post‐transcriptional level, by cleaving their mRNA. Because these target genes are mainly regulatory genes, miRNAs are involved at the crossroads of several biological processes (Liu *et al*., 2018). miRNAs are transcribed as long primary transcripts (pri‐miRNAs) (Xie *et al*., 2005). Recent findings revealed that plant pri‐miRNAs encode regulatory peptides called miRNA‐encoded peptides (miPEPs) (Lauressergues *et al*., 2015; Sharma *et al*., 2020). miPEPs specifically enhance the transcription of their pri‐miRNA, leading to phenotypes consistent with the functions of their cognate miRNA. Only a few miPEPs have been found as useful tools in agronomy. Indeed, soybean miPEP172c was shown to increase nodulation, aiming for a better yield (Couzigou *et al*., 2016), and grapevine miPEP171d increased adventitious root formation (Chen *et al*., 2020). In both cases, the role of the corresponding miRNA was previously known. Here, we questioned whether it would be feasible to screen a whole set of miPEPs for a particular phenotype in *Arabidopsis thaliana*, and whether it was possible to transpose these data to plants of agronomic interest.

We focused our study on the identification of *A. thaliana* miPEPs modulating root development. In the perspective to transpose our results from *A. thaliana* to other species of agronomic interests, we narrowed our analysis on miRNAs present in most plant species (conserved miRNAs). The first step consisted of identifying *A. thaliana* pri‐miRNAs, by crossing the data from EST sequences (www.ncbi.nlm.nih.gov/genbank/), Illumina RNA‐seq data (Wang *et al*., 2019) and RACE‐PCR data (Xie *et al*., 2005). Several examples previously established that the first ORF after the transcription start site corresponded to a translated ORF coding a miPEP able to increase pri‐miRNA expression (Chen *et al*., 2020; Couzigou *et al*., 2016, 2017; Lauressergues *et al*., 2015; Sharma *et al*., 2020; Zhang *et al*., 2020). Therefore, we selected the first ORF of each identified pri‐miRNA as a high confident candidate to produce functional miPEPs and synthesized the 87 miPEPs corresponding to the 87 conserved *A. thaliana* miRNAs, from miR156a to miR399f. We used an *in vitro* assay to monitor seedling root development after peptide treatments by measuring total root development. As controls, we used water, 0.5% acetonitrile and an irrelevant peptide, consisting of a random 10‐amino acid peptide showing no blast hit in any of the available plant genomes. In these conditions, we identified 23 miPEPs significantly modulating total root development, among 87 tested (Fig. 1a). Interestingly, most of the 23 miPEPs had an inhibitory effect on root growth without showing any apparent toxicity.

**Figure 1:**
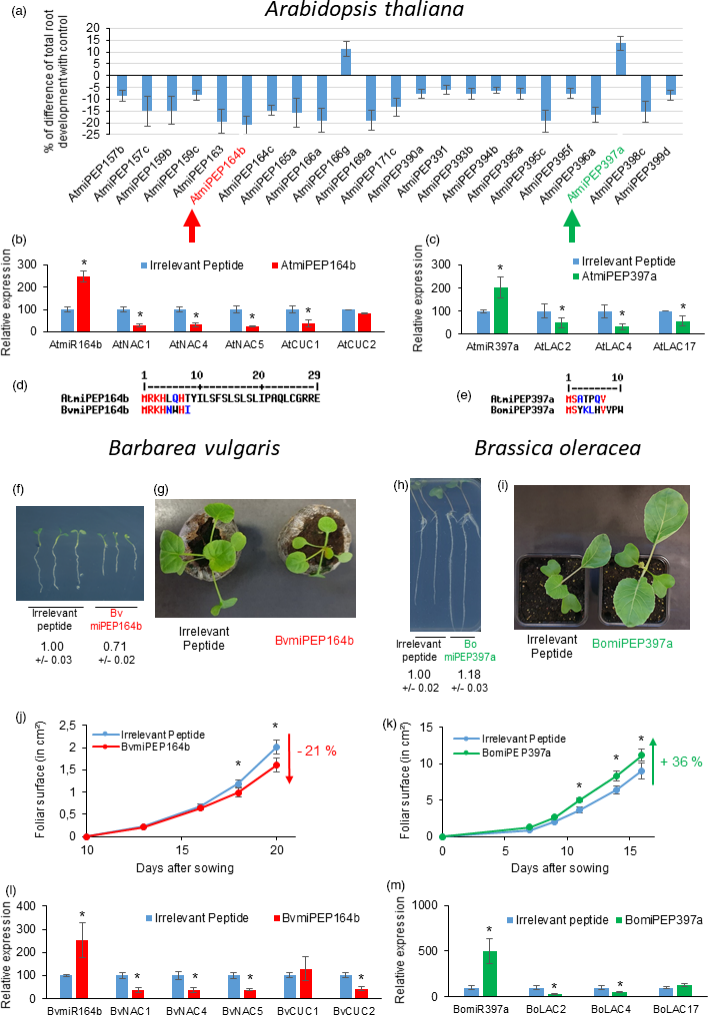
(a) Percentage of difference of total root development between *A. thaliana* plants treated with an irrelevant control peptide and each miPEP. Only miPEPs with significant effects according to the Student *T*‐test are shown. Expression of AtmiR164b (b) or AtmiR397a (c) and their target genes after treatment with irrelevant peptide or AtmiPEP164b or AtmiPEP397a. (d) Sequence alignment of miPEP164b from *A. thaliana* (AtmiPEP164b) with miPEP164b from *Barbarea vulgaris* (BvmiPEP164b). (e) Sequence alignment of miPEP397a from *A. thaliana* (AtmiPEP397a) with miPEP397a from *Brassica oleracea* (BomiPEP397a). (f) Root phenotype of *B. vulgaris* seedlings treated with BvmiPEP164b or irrelevant peptide. (g) Picture of *B. vulgaris* plants treated with BvmiPEP164b or irrelevant peptide, 21 days after sowing. (h) Root phenotype of *B. oleracea* seedlings treated with BomiPEP397a or irrelevant peptide. (i) Picture of cabbage plants treated with BomiPEP397a or irrelevant peptide, 19 days after sowing. Evolution of foliar surface of *B. vulgaris* (j) or cabbage (k) plants treated with BvmiPEP164b or BomiPEP397a or irrelevant peptide. Expression of BvmiR164b (l) or BomiR397a (m) and their target genes after treatment with irrelevant peptide or BvmiPEP164b or BomiPEP397a. Error bars represent SEMs, asterisks indicate a significant difference between test conditions and controls according to Wilcoxon test (b, c, l, m) or Student’s *T*‐test (a, f, h, j, k) (a: *n* = 120, b, c, l, m: *n* = 6. f–k: *n* = 60; *P* < 0.05). (f, h) Numbers under each image represent the mean value ± SEM of root development, normalized to the respective control (irrelevant peptide) set to 1.

Among these, we focused our attention on the two miPEPs with the strongest effect on growth, together with the best statistical *P*‐value: AtmiPEP164b and AtmiPEP397a as an inhibitor and an activator of root growth, respectively (Fig. 1a). We checked that these miPEPs were able to increase the expression of their nascent pri‐miRNAs and, correlatively, decrease the expression of the respective miRNA target genes (Fig. 1b, c).

MiRNAs are very well conserved among species, but not miPEPs (Couzigou *et al*., 2017; Lauressergues *et al*., 2015). In order to identify homologs of AtmiPEP164b and AtmiPEP397a in Brassicaceae of agronomic interest, we searched the corresponding pre‐miRNA on the genomes of the wild cabbage *Brassica oleracea*, and *Barbarea vulgaris*, a weed, which can be a seed contaminant (MacDonald and Cavers, 1991). We were able to identify only one miRNA homolog of each in the corresponding genome, and we next searched the corresponding miPEPs in these pre‐miRNAs and defined miPEPs in *B. oleracea* and *B. vulgaris* by the identification of the first ORF (Fig. 1d, e). We next cultivated *B*. *oleracea* and *B. vulgaris* seedlings *in vitro,* treated or not with the corresponding miPEP and monitored their root development. *Barbarea vulgaris* BvmiPEP164b was able to decrease root development (Fig. 1f) while *B. oleracea* BomiPEP397a was able to increase cabbage root development (Fig. 1h), showing that miPEPs exhibit a remarkable functional conservation of their function between species.

The overall development of a plant is closely subordinated to root development, and the modulation of root growth directly affects the development of aerial parts accordingly. So, we tested the effect of BomiPEP397a and BvmiPEP164b on cabbage and *B. vulgaris* growth, respectively. We found that watering *B. vulgaris* plants with BvmiPEP164b led to a 21% decrease in the foliar surface (Fig. 1g, j), while watering cabbage plants with BomiPEP397a led to a 36% increase in the foliar surface (Fig. 1i, k). Finally, we validated that BvmiPEP164b and BomiPEP397a were able to increase the expression of their respective pri‐miRNA while decreasing the expression of the corresponding target genes (Fig. 1l, m).

The identification of a whole set of miPEPs in certain plant species can be difficult due to the lack of strong genomic and transcriptomic data. We show here that a screening of a high number of miPEPs in *A. thaliana* followed by the identification of homologs in plants of interest is an efficient method to identify miPEPs active on a particular phenotype. In that way, we show that watering plants with miPEPs which modulate root growth can lead to an overall change in plant development, thus being a suitable alternative to the use of chemicals in agronomy.

Weeds are one of the major problems of world agriculture. While some countries try to strongly restrict the use of chemicals, more and more plants are starting to be resistant to these chemicals. Since miPEPs are very specific (Lauressergues *et al*., 2015), we can imagine the use of a cocktail of several peptides to improve the development of crops and their resistance to stresses (pathogens, starvation…) while reducing weed growth.

## Conflict of interest

Authors declare no conflict of interest.

## Author contributions

JPC designed the research; JPC, BG and MO performed the molecular biology and plant experiments; HSC performed bioinformatics analysis; BG analysed screening data; JPC, PT and SP wrote the paper.
